# Robotic Adrenalectomy: An Initial Experience in a Turkish Regional Hospital

**DOI:** 10.3389/fsurg.2022.847472

**Published:** 2022-06-29

**Authors:** Ayhan Erdemir, Kemal Rasa

**Affiliations:** Department of General Surgery, Anadolu Medical Center Hospital Kocaeli, Kocaeli, Turkey

**Keywords:** adrenalectomy, laparoscopic adrenalectomy, robotic adrenalectomy, adrenocortical cancer treatment, adrenal adenoma surgery

## Abstract

**Background:**

Due to the technical advantages and the convenience it provides to surgeons, “robotic adrenalectomy” is a widely used procedure for adrenal surgeries. In this study, we aim to evaluate our robotic adrenalectomy experience and delineate the factors that have a substantial impact on surgical outcomes.

**Methods:**

Successive 0 transperitoneal robotic operations using the daVinci SI^®^ platform were grouped according to the surgery side, malignant or benign pathologies, for adenoma or non-adenoma lesions, tumor size of less than 4 cm or above, body mass index below or above 30 kg/m^2^, and with or without laparotomy history. Groups were compared in terms of duration of the operations, amount of bleeding, and the duration of hospitalization.

**Results:**

Morbidity developed in 5 patients (16.6%), and no mortality was observed. We had only one conversion to perform open surgery (3.3%). Operations performed for adenoma significantly last longer when compared with the non-adenoma group (*p* < 0.05). In the malignant group, the amount of bleeding during surgery was found to be significantly higher (*p* < 0.05). The blood loss during the surgery was also found to be higher in the adenoma group than in the non-adenoma match (*p* < 0.05). Phenomenally, operative blood loss was found to be lesser in the bigger tumor size group (>4 cm) than in the smaller size group (*p* < 0.05).

**Conclusion:**

Our results corroborate that robotic adrenalectomy may be more challenging for malignant pathologies and adenomas, but we can claim that it is an effective and safe option for all adrenal gland pathologies.

## Introduction

In recent years, surgeries using robotics are being preferred over open and laparoscopic operations within various masteries. The transition from open surgery to laparoscopy and then to robotic surgery has provided many advantages to both patients and surgeons. The advantages include shorter hospital stays, less pain, lower risk of surgical site infection, less blood loss and transfusion requirement, and less scar tissue development. In addition to faster recovery and return to work and normal life, the advantages of laparoscopic surgery, the three-dimensional vision, 12-fold magnification effect, comfortable wrist movements, and the absence of shakiness during robotic surgery have all brought adrenal gland surgery to a whole new dimension.

For a vast majority of adrenal gland pathologies, adrenalectomy is the main treatment modality (1). For many years, open adrenalectomy was accepted as the gold standard. Laparoscopic adrenalectomy, first performed by Gagner in 1992, was performed in many centers in the following years, and due to successful results, has now become the standard technique (2, 3). The interest in the “robotic adrenalectomy” option has increased in recent years due to the technical advantages ensured by robotic technology and the ease it provides to the surgeon. Existing laparoscopy experiences of surgeons have made it easier and faster to adapt to robotic adrenalectomy. Numerous studies have proven that robotic adrenalectomy is an effective and safe modality and can be the best option for adrenal gland surgery (4, 5).

In this study, we aim to evaluate the robotic adrenalectomy operations performed in a single center by assessing not only the operation process but also the factors that affect the outcomes of those operations.

## Materials and Method

The Anadolu Medical Center Hospital has been using robotic surgery since 2011. The General Surgery team at AMC took a leading part to implement this technology to clinical practice. In this study, 30 successive robotic adrenalectomy operations are evaluated, and the break-up is as follows: 3 cases in 2011, 5 in 2012, 3 in 2013, 3 in 2014, 2 in 2015, 2 in 2016, 2 in 2017, 1 in 2018, 3 in 2019, 3 in 2020, and 3 in 2021.

The surgeries were executed by a team with an advanced laparoscopic surgery experience as well as certifications on robotic surgery. Apart from demographic characteristics, patients were divided into groups based on the following: surgery side, malignant or benign, ones with adenoma and non-adenoma, those with a tumor size of less than 4 cm and above, those with a body mass index (BMI) below or above 30 kg/m^2^, and those with and without laparotomy history. The duration of surgery, amount of bleeding, and hospital stay were evaluated in each group.

All surgeries were done transperitoneally using the da Vinci SI^®^ robot platform. Only three robot arms were used after the first three surgeries. Patients were positioned at the operating table, at a standard 45° lateral decubitus, and a 12 mm trocar and a camera were placed after the pneumoperitoneum at a pressure of 12 mmHg created *via* the Veress needle. For 21 patients, we used one trocar (10 mm) as an assistant port, and for 9 patients, we inserted a second assistant port. Two 8 mm trocars for robot arms were also inserted. A separate 5 mm port was inserted to be used for liver retraction in patients who underwent right adrenalectomy. Caution was exercised to ensure that the camera port was 20 cm away from the target organ and to keep at least 7 cm between the ports so that the robot arms do not overlap. The robot was brought closer to the operating table from the patient’s right or left shoulder area. We have carefully ensured that the camera port, the target organ, and the camera arm of the robot were on the same axis. After connecting the robot arms with the ports, the operation was performed with a method similar to laparoscopic surgery.

The study was conducted in accordance with the Declaration of Helsinki (as revised in 2013), and individual consent for this retrospective data analysis was waived, because for the studies based on retrospective data/file analysis, the Anadolu Medical Center Review Board and Ethics Committee waived the need for ethics approval.

## Statistical Evaluation

In the descriptive statistics of the data, mean, standard deviation, minimum and maximum median, frequency, and ratio values were used. The distribution of variables was measured with the Kolmogorov Smirnov test. The Mann–Whitney *U* test was used in the analysis of quantitative independent data. The SPSS 27.0 program was used in the analysis.

## Results

The study included 13 men and 17 women with a median age of 46.4 years (Table 1). Nineteen of the surgeries were performed for right adrenal pathologies and 11 for left adrenal pathologies. Eleven of the patients were operated for malignant tumors, while 19 of them were operated for benign tumors (Table 2). While the number of patients diagnosed with adenoma was determined as 9, 21 patients were operated for reasons other than adenoma. The tumor size of 3 patients was below 4 cm and 27 patients had tumors that exceeded 4 cm. While the number of patients with a BMI below 30 was 12, it was calculated as above 30 in 18 patients. While 10 patients with a history of laparotomy were included in the study, 20 patients did not have a history of surgery.

**Table 1:** Demographic characteristics of 30 evaluated patients.

Median age	46.4 years
Gender	Female: 17
	Male: 13
Side	Right: 19
	Left: 11
Mean duration of hospitalization	3.5 days
Mean blood loss	226.5 ml
Mean anesthesia time	194 min
Mean BMI	30.1 kg/m^2^
Mean tumor size	8.3 cm
Conversion to open surgery	1 (3.3%)
Morbidity	5 (16.6%)
Mortality	0
Readmission	2 (6.6%)

**Table 2:** Adrenal gland pathologies of 30 evaluated patients.

Pathology	Number of patients
Adenoma	10
Malignancy	
Pheochromocytoma	5
Carcinoma	3
Lung cancer metastasis	2
Malignant melanoma metastasis	1
Adrenocortical neoplasm	1
Ganglioneuroma	2
Cushing's syndrome	2
Nonneoplastic lesion with hemorrhage	2
Granulomatous disease	1
Hyperplasia	1

The mean duration of anesthesia was 194.8 min. Mean blood loss was 226.5 ml, and hospital stay was 3.5 days. The mean tumor size was 8.3 cm, and the mean BMI was calculated as 30.1 kg/m^2^.

Morbidity developed in five patients (16.6%), and all such events were rated as “Grade 1” according to the Dindo Clavien score. Two patients had postoperative fever and one patient had atelectasis. We had one patient with a superficial surgical site infection. One patient had high blood pressure during the postoperative period, and it was necessary to use multiple antihypertensive medications to handle the situation. There was one conversion to open surgery (3.3%), but no mortality was observed.

Six different analyses were performed based on six different criteria. These criteria were the location of the pathology, whether it is a malignant or benign disease, whether it is due to an adenoma or a non-adenoma, whether the tumor size was less than 4 cm or greater than 4 cm, patients with a BMI below or above 30 kg/m^2^, and patients with a history of laparotomy or without any history of surgery. The groups were compared according to the duration of surgery, amount of bleeding, and the duration of hospitalization.

Performing the operation for the right or left adrenal gland did not significantly change the duration of the surgery (*p* > 0.05). There was no significant difference between the malignant and the benign groups in terms of the duration of the operation as well (*p* > 0.05). It was observed that operations performed for adenomas significantly lasted longer than the surgeries performed for non-adenoma reasons (*p* < 0.05). Tumor size was found to have no impact on the duration of the surgery (*p* > 0.05). It was also observed that a BMI below or above 30 kg/m^2^ and a history of laparotomy had no impact on the duration of surgery (*p* > 0.05) (Table 3).

**Table 3:** Duration (minutes) of the robotic adrenalectomy operations according to the analyzed criteria.

* *	*n*	Min–Max (min)	Median (min)	Mean ± ss (min)	*p*
Total	30	100–480	180	194.9 ± 80.1	
Side					
Right	19	100–300	180	182.2 ± 61.4	0.388^m^
Left	11	100–480	200	216.8 ± 104.9	
Malignancy status					
Benign	19	115–300	195	206.0 ± 67.0	0.320^m^
Malignant	11	100–480	180	188.4 ± 87.9	
Adenoma					** **
Non-adenoma	21	100–207	150	152.7 ± 34.6	**0.041^m^**
Adenoma	9	100–480	195	213.0 ± 87.7	
Size					
<4 cm	3	100–480	180	192.9 ± 83.8	0.331^m^
>4 cm	27	180–250	207	212.3 ± 35.3	
BMI					
<30 kg/m^2^	18	100–273	158.5	176.1 ± 61.5	0.251^m^
>30 kg/m^2^	12	100–480	183.0	207.4 ± 90.0	
Laparotomy history					
Present	10	100–250	187.5	188.4 ± 47.7	0.808^m^
Absent	20	100–480	180	198.1 ± 93.2	

***P:*** *^m^Mann–Whitney U test*.

Performing the surgery for the right or left adrenal gland did not change the amount of bleeding (*p* > 0.05). The amount of bleeding that occurred during the operations of the patients in the malignant group was found to be significantly higher than that in the benign group (*p* < 0.05). The amount of bleeding in the operations performed for adenoma was higher than that for the group operated for non-adenoma (*p* < 0.05). The amount of bleeding was found to be significantly higher in the group whose tumor size was less than 4 cm compared with the group whose tumor size was larger than 4 cm (*p* < 0.05). When the groups with BMI below 30 kg/m^2^ and above 30 kg/m^2^ were compared, it was found that the amount of bleeding did not change significantly (*p* > 0.05). When the groups with and without a history of laparotomy were compared, there were no significant difference in the blood loss (*p* > 0.05) (Table 4).

**Table 4:** Bleeding amount (ml) during surgery according to the analyzed criteria.

* *	*n*	Min–Max (ml)	Median (ml)	Mean ± ss (ml)	*p*
Total	30	0–2,300	100	226.5 ± 426.4	
Side					
Right	19	0–600	100	172.4 ± 188.4	0.983^m^
Left	11	0–2300	100	320.0 ± 669.6	
Malignancy status					
Benign	19	50–500	150	225.0 ± 163.2	**0.028^m^**
Malignant	11	0–2300	50	227.4 ± 527.4	
Adenoma					
Non-adenoma	21	0–600	50	113.3 ± 190.0	0.104^m^
Adenoma	9	0–2300	100	275.0 ± 490.9	
Size					
<4 cm	3	0–2300	100	249.8 ± 444.0	**0.025^m^**
>4 cm	27	0–50	0	16.7 ± 28.9	
BMI					
<30 kg/m^2^	18	0–350	100	126.7 ± 86.3	0.652^m^
>30 kg/m^2^	12	0–2300	75	293.1 ± 541.9	
Laparotomy history					
Present	10	0–500	100	130.0 ± 141.8	0.656^m^
Absent	20	0–2300	100	274.8 ± 510.6	

***P:*** *^m^Mann–Whitney U test.*

When the duration of hospitalization was evaluated, no statistically significant difference was observed between right and left adrenal surgeries, malignant and benign pathologies, adenoma and non-adenoma causes, groups with a tumor size below and above 4 cm, groups with a BMI below and above 30 kg/m^2^, and groups with and without a history of laparotomy (*p* > 0.05) (Table 5).

**Table 5:** Hospital stay (days) for robotic adrenalectomy surgery according to the analyzed criteria.

* *	*n*	Min–Max (days)	Median (day)	Mean ± ss (days)	*p*
Total	30	1–8	3.0	3.50 ± 1.89	
Side					
Right	19	1–7	3.0	3.53 ± 1.87	0.771^m^
Left	11	2–8	2.0	3.45 ± 2.02	
Malignancy status					
Benign	19	1–7	2.0	3.09 ± 1.81	0.304^m^
Malignant	11	2–8	3.0	3.74 ± 1.94	
Adenoma					
Non-adenoma	21	2–7	3.0	4.00 ± 2.12	0.397^m^
Adenoma	9	1–8	3.0	3.29 ± 1.79	
Size					
<4 cm	3	1–8	3.0	3.48 ± 1.91	0.829^m^
>4 cm	27	2–6	3.0	3.67 ± 2.08	
BMI					
<30 kg/m^2^	18	1–7	2.0	3.08 ± 1.78	0.253^m^
>30 kg/m^2^	12	2–8	3.0	3.78 ± 1.96	
Laparotomy history					
Present	10	2–7	3.0	3.70 ± 1.77	0.493^m^
Absent	20	1–8	2.5	3.40 ± 1.98	

***P:*** *^m^Mann–Whitney U test.*

## Discussion

The first successful robotic surgery was a fundoplication performed by Cadiere et al. in 1997, and the first robotic adrenalectomy was performed in 2001 (6, 7). Besides open surgery, adrenal gland operations can be performed with laparoscopic transabdominal, laparoscopic retroperitoneal (retroperitoneoscopic), robotic transabdominal, and robotic retroperitoneal approaches. Despite the existence of these options, the laparoscopic transabdominal approach is still accepted as the gold standard (8). Open surgery may still be recommended in selected patient groups, for instance in patients who have adrenal gland cancer whose tumor size is larger than 8 cm (9). In the study published by Assalia et al., in which open and laparoscopic surgeries were compared, the laparoscopic approach was shown to be superior even in tumors of 10–12 cm in size (10). Different results have also been reported for retroperitoneoscopic adrenalectomy. While some studies have shown that shorter operation and hospitalization times can be achieved with this method, some studies have concluded that this approach does not shorten these durations, and there is no difference between techniques (11–13).

The absence of a feeling of tension in robotic instruments, difficulties in changing instruments, and the lack of difference in the length of hospital stay also discourage the robotic option (14). In a study conducted by Morino et al., laparoscopic and robotic surgeries were compared, and it was reported that the duration of the operation was longer in the robotic group, and 10 conversions to laparoscopic surgery were reported (15). In this study, in which hospitalization durations were similar in both groups, 20% morbidity was found in the robotic surgery group, while there was no morbidity in the laparoscopic group (15). In another study by Bround et al., 50 robotic and 59 laparoscopic adrenalectomies were compared, and it was reported that blood loss in robotic adrenalectomy group was lower. Following the completion of the learning curve, it was stated that the duration of surgery was similar to that of the laparoscopic surgery group (16). It was observed that the duration of the operation was prolonged in the laparoscopic group if the BMI was above 30 kg/m^2^, but a high BMI had no impact on surgery time in the robotic group (16).

Growing data on robotic adrenalectomy for resectable adrenal malignancies encourage surgeons to prefer the robotic approach. In line with this, Hue at al. recently reported their planned minimally invasive adrenalectomy cohort for adrenal malignancies (adrenocortical carcinoma, malignant pheochromocytoma, and other carcinoma) (17). A total of 416 patients (76.5%) underwent a laparoscopic adrenalectomy and 128 (23.5%) underwent a robotic operation. The conversion rate decreased among robotic adrenalectomies relative to laparoscopy on univariate (7.8% vs. 18.3%, *p* = 0.005) and multivariable (odds ratio 0.39, *p* = 0.01) analyses. They concluded that in contrast to most clinical guidelines, minimally invasive adrenalectomies are being performed on large malignant tumors. They also stated that the laparoscopic approach was associated with a greater conversion rate and subsequent poor outcomes. Calcetera et al. evaluated 588 patients who underwent adrenalectomy for adrenocortical carcinomas, of which 200 were minimally invasive (18). From 2010 to 2014, minimally invasive operations increased from 26% to 44%, with robotic procedures increasing from 5% to 16%. They concluded that the frequency of minimally invasive approaches for adrenocortical carcinomas was increasing, and in the final year of the study, 44% of adrenalectomies were found to be minimally invasive.

In this study, we aim to evaluate our initial 30 transperitoneal robotic adrenalectomy experiences and delineate the factors that have a substantial impact on surgical outcomes.

Our study has explicit limitations, notably the low number of patients. The commonly held number of patients to complete the learning curve for robotic adrenalectomy for a center is 20, and our experience exceeds this limit only by 10 patients. We are also aware that statistical analysis with this limited number of patients allows us to draw only relative conclusions. We also think that we would have much more to say if we had a control group of patients who underwent open or laparoscopic adrenalectomy that we could compare with this robotic adrenalectomy cohort.

At first, our experience in these surgeries taught us the gravity of the alignment of the robotic arms and the ease it provides to the operation's overall success. An overlap negatively affects the operation time and the total amount of bleeding. This can be a serious problem, especially with slim patients. Although the problem is less frequent with three robotic arms, we must be careful during the trocar placement phase at the beginning of each surgery. There must be a distance of at least 7 cm between the robotic arms, and this distance must be secured for the vertical axis as well. While this distance is maintained transversely, not paying attention to this distance in the vertical axis can also lead to issues during the operation. This problem can be eliminated by maneuvering the arms of the robot on the abdominal wall. Even if the problem is resolved during the operation, these traumatic maneuvers on the abdominal wall may cause port site problems.

In our study, the mean duration of the operation was 194.8 min, which seems to remain stable over the years. Similar studies evaluating the learning curve for robotic adrenalectomy stated that the duration of the operation decreased significantly after the first 20 operations (16, 19), and these data lend credence to our assumption that we still have room for improvement for operation time in the future. Among analyzed parameters, it was observed that operations performed for adenomas lasted longer when compared with the surgeries performed for non-adenoma. Since a significant number of these surgeries, 6 out of 9, were performed in the very first years of our robotic surgery experience, the “learning curve effect” may be a crucial factor, although, due to the low number of cases, the impact of the “learning curve effect” could not be accurately evaluated.

The mean blood loss was found to be 226 ml in our study. The amount of bleeding that occurred during the operations performed for malignant pathologies were found to be higher when compared with the operations for benign pathologies. This finding can be explicated with an increased vascularization of these lesions and technical difficulties of cancer surgeries. Higher intraoperative blood loss in patients with tumor size less than 4 cm when compared with its bigger counterpart (>4 cm) was indeed an unpredictable data. Our limited sample size, with the presence of only 3 patients of this group (<4 cm), may have an effect on this outcome, and we concluded that we need to have a prospective study with a much higher number of patients to define the impact of different parameters, notably the size on operative blood loss.

When the duration of hospitalization was evaluated, no statistically significant difference was observed between right and left adrenal surgeries, malignant and benign pathologies, adenoma and non-adenoma causes, groups with a tumor size below and above 4 cm, groups with a BMI below and above 30 kg/m^2^, and groups with and without a history of laparotomy as expected.

Another remarkable finding of our study is that tumor size, obesity, and history of previous abdominal surgery did not affect the duration of surgery, amount of bleeding, and hospital stay.

The impact of tumor size on operation time was evaluated by Bround et al., and it was observed that the operation time was prolonged in the laparoscopic group for tumors larger than 55 mm. However, there was no change in terms of time in the robotic group (20). Our study found no statistically significant difference in terms of operation time, amount of bleeding, and hospital stay in patients with tumors greater than 4 cm, which supports our claim that robotic surgery can be used safely in this group of patients.

Besides larger tumors, Giulianotti et al. reported that robotic adrenalectomy is a feasible option for obese patients (20). In another study evaluating obesity and comparing 42 robotic and 57 laparoscopic adrenal surgeries, the operation time, blood loss, and hospital stays were found to be similar in both groups. It has been concluded that the ability to angle the robotic instruments provides a significant advantage in obese patients compared with laparoscopic surgery that uses rigid instruments (21). Our duration of surgery, amount of bleeding, and hospital stay in high BMI patients, above 30 kg/m^2^, were similar compared with those in low BMI patients. Hence, we can say that a high BMI should not be a discouraging issue for the robotic option.

We have also found that the other two parameters, the side of the surgery and laparotomy history, have no impact on the duration of surgery, the amount of intraoperative blood loss, and hospital stay.

## Conclusion

The results of our initial robotic adrenalectomy experience indicated that the operations for adenomas may last longer and surgeries for malignant cases may cause higher blood loss, but nevertheless, we believe that our results make a solid contribution to the view that robotic adrenalectomy is an effective and safe option for all adrenal gland pathologies. To reduce and even get rid of the learning curve effect and confirm our conclusion, we need to have a prospective, nationwide, multicenter study with a much higher number of patients.

## Data Availability

The original contributions presented in the study are included in the article/Supplementary Material; further inquiries can be directed to the corresponding author/s.
